# Orphanet Journal of Rare Diseases: Launch Editorial

**DOI:** 10.1186/1750-1172-1-1

**Published:** 2006-03-01

**Authors:** Ségolène Aymé, Bruno Dallapiccola, Dian Donnai

We are pleased to announce the launch of *Orphanet Journal of Rare Diseases*, an open access, peer-reviewed journal that will encompass all aspects of rare diseases and orphan drugs. The focus will be on review articles, but the journal will consider all contributions that are scientifically sound and within its scope.

We wish to express our gratitude to the many colleagues who have agreed to serve as section editors for their specialties. To a considerable extent, the quality of the journal will depend on their commitment. The journal's editorial office is established at the Orphanet headquarters in Paris and supported by several major institutions including INSERM, the French Ministry of Health and the European Commission (DG Public Health and Consumer Protection).

Traditionally, readers pay to access articles, either through subscriptions or by paying a fee each time they download an article. Escalating costs of journal subscriptions have resulted in libraries subscribing to fewer journals and the range of articles available to readers is therefore increasingly limited. Although traditional journals publish authors' work for free (unless there are page or colour charges), there are limits on how many people can read, use and cite these articles given the requirement to pay for access.

*Orphanet Journal of Rare Diseases' *open access policy changes the way in which articles are published. First, all articles become freely and universally accessible online, and so an author's work can be read by anyone at no cost. Second, the authors hold copyright for their work and grant anyone the right to reproduce and disseminate the article, provided that it is correctly cited and no errors are introduced. Third, the results of publicly funded research will be accessible to all taxpayers and not just those with access to a library with a subscription. As such, open access holds the potential to increase public interest in, and support of, research. Fourth, a country's economic strength will not influence its researchers' ability to access articles because readers in resource-poor countries (and institutions) will be able to access the same material as wealthier ones (access to the internet being the only requirement).

Article-processing charges will enable *Orphanet Journal of Rare Diseases *to serve the scientific community with this open access forum. We hope you will support this major advance by submitting articles to our journal. *Orphanet Journal of Rare Diseases *will usually levy an article-processing charge for manuscripts accepted after peer review (payable on acceptance), but will cover the cost of publication for invited review articles. Many institutions [1] and research funders [2] will cover article-processing charges for their researchers.

Please submit your work to *Orphanet Journal of Rare Diseases *[3]. Help us to contribute to increasing knowledge on rare diseases for the benefit of the patients and their families and to assist professionals and researchers working in the field.

Ségolène Aymé, Bruno Dallapiccola and Dian Donnai

The Editors-in-Chief, *Orphanet Journal of Rare Diseases*
